# Adaptation to altered balance conditions in unilateral amputees due to atherosclerosis: a randomized controlled study

**DOI:** 10.1186/1471-2474-12-118

**Published:** 2011-05-27

**Authors:** Ágnes Mayer, József Tihanyi, Károly Bretz, Zsolt Csende, Éva Bretz, Mónika Horváth

**Affiliations:** 1 St. George University Teaching Hospital, Physiotherapy, 8000 Székesfehérvár, Seregélyesi u. 3, Hungary; 2 Semmelweis University, Faculty of Physical Education and Sport Sciences, 1122 Budapest, Alkotás u. 144, Hungary; 3 Semmelweis University, Faculty of Health Sciences Hungary, 1088 Budapest, Vas u. 17, Hungary

## Abstract

**Background:**

Amputation impairs the ability to balance. We examined adaptation strategies in balance following dysvascularity-induced unilateral tibial amputation in skilled prosthetic users (SPU) and first fitted amputees (FFA) (N = 28).

**Methods:**

Excursions of center of pressure (COP) were determined during 20 s quiet standing using a stabilometry system with eyes-open on both legs or on the non-affected leg(s). Main measures: COP trajectories and time functions; distribution of reaction forces between the two legs; inclination angles obtained through second order regression analysis using stabilogram data.

**Results:**

FFA vs SPU demonstrated 27.8% greater postural sway in bilateral stance (p = 0.0004). Postural sway area was smaller in FFA standing on the non-affected leg compared with SPU (p = 0.028). The slope of the regression line indicating postural stability was nearly identical in FFA and SPU and the direction of regression line was opposite for the left and right leg amputees.

**Conclusion:**

Of the two adaptation strategies in balance, the first appears before amputation due to pain and fatigue in the affected leg. This strategy appears in the form of reduced postural sway while standing on the non-affected leg. The second adaptation occurs during rehabilitation and regular use of the prosthesis resulting in normal weightbearing associated with reduced postural sway on two legs and return to the normal postural stability on one leg.

## Background

There is a high incidence of peripheral vascular disease (PVD) in old adults. Atherosclerosis and chronic ischemia are the main causes of PVD in the lower extremities. Symptoms of PVD include pain and fatigue while standing and walking and there is also a concomitant reduction in leg strength [1]. PVD is thus associated with impaired lower extremity function and postural stability [2,3]. For example, Suominen et al. [4] recently reported that PVD patients compared with aged matched healthy controls had significantly higher postural sway velocity in anteroposterior and mediolateral directions.

Curiously, however, there is a paucity of data on patients with unilateral lower leg PVD whether these patients would adapt their postural stability before surgery. The presence of an adaptive strategy in balance before surgery is likely because such pre-surgery changes have been proposed to persist during the prosthetic fitting period following surgery. Except in one study [5], several studies reported that postural stability was impaired after surgery in skilled prosthetic users [6-12] as well as first fitted amputees [13-15]. Specifically, there is excessive lateral postural sway and there are also larger than expected excursions in the center of pressure (COP) in the anteroposterior direction in due to an increase in weight-bearing on the non-affected leg [7,14]. Weight-bearing asymmetry interferes with postural stability and forces even healthy adults to modify their postural control strategy [16] and such modifications have been observed in patients with neuromuscular disorder [17-19]. A recent dynamic stabilometry study concluded that below-knee amputees placed more load on their non-affected leg and also performed larger movement and that this strategy helped these patients to minimize postural stability disturbances [12].

One would predict that loading the non-affected limb during double compared with single leg stance would tend to decrease postural sway yet there is no significant relationship between postural performance measured using one and two legged stance [20], suggesting that the balance control mechanism differs between standing on two vs. one leg [21,22]. This suggestion may be supported by the result of Mak and Ng [23] reporting significantly decreased postural sway standing on single leg in people practicing Tai-Chi. In fact nobody has studied this problem explicitly so far on first fitted and experienced unilateral vascular amputees despite the fact it has been shown that postural sway on single leg is a useful indicator for the incident of falls [24,25]. Indeed, in the only study so far, Hermodsson et al. reported significant difference between experienced amputees and healthy controls in postural sway while standing on both legs but not when standing on one the affected leg [10].

Considering the inconsistencies in the literature concerning COP behaviour while standing on one vs. two legs in amputees, here we examine the possibility that postural balance adaptations differ between first fitted amputees and skilled prosthesis users. Our working hypothesis was that vascular insufficiency in the affected leg had induced some degree of weight-bearing asymmetry long before amputation, organising postural control around the non-affected side. Specifically we hypothesized that the magnitude of postural sway is reduced in unilateral standing due to the favouring of the intact leg during this initial period. We also examined the possibility that regular use of the prosthesis during the prosthetic fitting period and following rehabilitation a second adaptation occurs, resulting in normal weight distribution characterized by reduced postural sway on two legs and return to the normal postural stability on single leg.

## Method

### Subjects

In the National Institute of Medical Rehabilitation Centre 21 patients met the main inclusion criteria, i.e., unilateral below knee amputation due to vascular disease and 18 patients (11 males, 7 females) met the secondary inclusion criteria, i.e., participation in regular physiotherapy and in walking practice (Table 1). All of them were amputated on minimum three maximum eight weeks prior to their starting to walk with the prosthesis and 2-4 days before the balance test.

**Table 1:** Clinical features and clinical assessment of amputees (Mean(SD)

Groups	Sex	Age	Body height (m)	Body mass (kg)	Barthel index	Time elapsed since amputation	Amputated side
Skilled prosthesis users (SPU)	8 male 2 female	61.1(10.5)	1.72(0.09)	82.9(17.2)	100(0)	4.15(2.4) years	8 right 2 left

First-fitted Amputees (FFA)	12 male 6 female	64.8(9.5)	1.64(0.1)	65.8(16)	67(4.8)	5.6(1.7) months	7 right 11 left

Members of the skilled prosthetic users group (SPU, n = 8M, 2F) underwent amputation 4.05 (±2.28) years before the start of the study and have been receiving regular physiotherapy and in walking practice (Table 1). Patients in the SPU group had already been wearing their prosthesis for 8-10 hours per day on average. Five patients from the original 18 withdrew from the testing for fear from standing on one leg and falling. Three of the remaining 13 withdrew because they were unable to stand for 20 s on one leg. The 10 patients who remained in the study used a prosthesis that was manufactured by the same company with a PTB-socket, and SACH-foot and a conventional cotton liner. The SPU patients used one stick during daily activity. The Barthel index was used to characterize patients' self-care and activity level (Table 1). Before the experiment the patients were informed about the measurements and possible risks involved in testing postural stability. The research and ethics committees of Semmelweis University, Budapest and the hospital approved the study. All patients gave written informed consent according to the Declaration of Helsinki.

### Instrumentation

**Figure 1:**
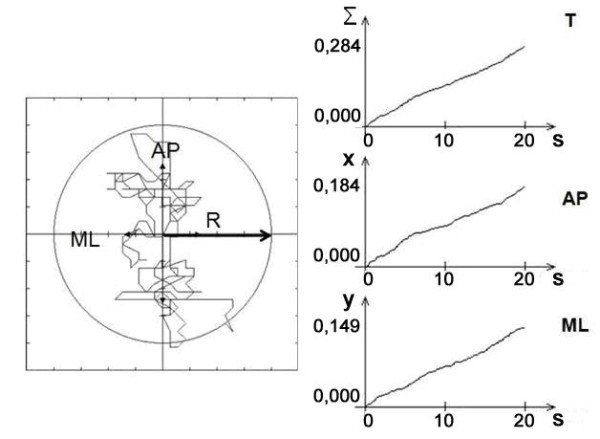
**Stabilograms**. Left: stabilogram indicating COP excursions and the radius of the characteristic circle containing 95% of the scanned points of the stabilogram. Right: total (T), AP and ML COP excursion lengths (m) in the function of the time during 20 s quite stance.

A stabilometer (ZWE-PII, Budapest) consisted of a force platform (size: 0.5m × 0.5m × 0.1m; range: 20 - 2000 N; linearity: ±1.5%; hysteresis: ±1.5%) amplifiers, a micro-computer, a PC, a monitor and custom software. COP data were sampled at 100 Hz. The dependent variables were COP trajectories and time functions, radius of the characteristic circle of the stabilogram containing 95% of its sampled points, total length of COP excursions; length of excursion in anteroposterior and mediolateral directions were visualized on the monitor (Figure 1).

### Data analysis

The following formula was applied to calculate the total path length of COP displacements:(1)

where *n *is the number of samples given by x_i_, y_i _coordinates, *i *is the numbering.

The procedure to compute the radius of the characteristic circle consists of the following steps:

1. computing the centre of gravity of the stabilogram P(x_c_, y_c_), determining the averages of the x_i _and y_i _values, which represent the stabilogram,

2. computing and saving distances of each sampled point of stabilogram: P(x_i_, y_i_), and the P(x_c_, y_c_), which is the centre if gravity of the stabilogram,

3. performing procedure "bubble sort" on the set of distances,

4. selecting the greatest distance value within the lower 95% subset of the computed set, consequently this length will be the radius magnitude of the characteristic circle, containing the inside 95% of sampled points of the stabilogram.

**Figure 2:**
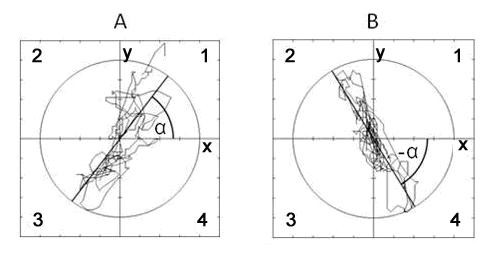
**Representative stabilograms indicating AP (y) and ML (x) excursions of COP**. A: COP excursion of a patient who had left lower leg amputation. The correlation coefficient is R_xy _= 0.75 (p <0.001). The regression line tends from the (left) prosthesis heel towards the right (intact) sole and the angle of regression line is  a = 53°. B: COP excursion of a right leg amputee. The correlation coefficient is: R_xy _= 0.72 (p <0.001) and the angle of regression line is    a = -48°. The regression line trends from the (right) prostheses heel towards the left intact sole. The quadrants of the coordinate system are marked with numbers: 1, 2, 3 and 4.

In order to identify the slope, i.e. the dominant direction of the COP movements of the amputees, the center of the stabilogram (x_c_, y_c_) was computed using the coordinates of the sampled points of the trajectories (x_i _y_i_) (Figure 2). This center was shifted to the origin of the coordinate system, which was displayed on the computer monitor, showing at the same time the middle of the force platform.

Then, we computed the correlation coefficient and performed a second order regression analysis on the stabilogram trajectory (equation 2, 3, 4)(2)

Where m_x _and m_y _are averages, s_x _and s_y _are standard deviations, R_xy _is the correlation coefficient.

The inclination angle (α) of the regression line was calculated as follows(3)

Equation 3 is an estimation of equation 1, from which the regression equation is computed.(4)

Where  and  are averages.

### Procedure

Postural Stability Test. During the test, patients wore light clothes and their own comfortable shoes on both feet with similar hell height. Participants were requested to stand on the platform with feet parallel at hip width, concentrating on the target located on the wall at a 2 m away at eye level. Recordings were made over a 20 s epoch. Participants had one or two practice trials to become familiar with the procedure. There were 2-3 minutes of rest between trials. For safety reasons, two walking bars were placed next to the patients spaced 0.7 m apart to prevent falling.

Postural stability was also measured while standing on one leg. Patients stood on the force platform in a double-leg stance, grabbing the walking bars, and then they shifted the weight to the intact leg. The other limb was lifted by flexing the knee and the hip, and the heel was next to the shank. Measurements were taken while standing on one leg with the arms in vertical position next to the thigh. Patients performed two trials with 2 to 3 minutes of inter-trial rest.

### Load Distribution (LD)

LD between the prosthesis and the intact limb was studied with eyes open only. Patients were asked to stand with a comfortable and stable erect posture as described under the Stabilometry test. The non-affected leg was placed on the force platform. The ground reaction force was recorded for 20 s. Average force was calculated for the intact leg (Fil) and this value was subtracted from the total body weight to obtain the force on the prosthetic leg (Fpl). The difference between Fil and Fpl was computed (LDi) and expressed in percentage.

### Statistical analysis

Descriptive statistics included mean and standard deviation (SD). The normality of all variables were checked with Shapiro-Wilk's test. The dependent variables were not normally distributed. Therefore to check for differences between groups nine Mann-Whitney U-tests were used with an adjusted p level of 0.05. Presumed differences between corresponding variables of the same group were examined by Wilcoxon-test. The relationship between the variables was computed by Spearman rank-correlation. The level of significance was set to p <0.05. To reveal the significance of those variables that describe the circle of the curve (R_D _and R_S_) in group classification a multivariate exploratory technique, discriminant function analysis was used with p <0.05 level. The standard method variable entering method was used with a tolerance level of 0.01, F to enter was set to 1. To detect partial or spurious correlation between the predictor variables that could influence the interpretation of the discriminant analysis the following hypothesis were tested.

Hr: There will be a correlation between R_S _and R_D_

H_0_: There will be no correlation between R_S _and R_D_

The data was processed with Statistica 8.0 version (S*tatSoft, Inc*., *Tulsa, OK, USA) *software.

## Results

The first fitted (FFA) patients' Barthel index was 67.8 (±4.3). Each skilled prosthesis user (SPU) scored the maximum 100 (Table 1).

### Postural Stability Test

Table 2 shows the results for the Postural Stability test.

**Table 2:** Balance test variables and load distribution for skilled prosthetic users (SPU) and for first fitted amputees (FFA), (Means(SD)

Variables and abbreviations	SPU (n = 10)	FFA (n = 18)	P value
Radius of the circle double leg (mm): R_D_	10.90 (5.3)	18.5 (6.6)	0.0014

Total excursion length double leg (mm): T_D_	468.3 (166.3)	662.6 (267.7)	0.071

Anteroposterior excursion length double leg (mm):AP_D_	331.1 (151.4)	398.9 (141.1)	0.145

Mediolateral excursion length double leg (mm):ML_D_	255.9 (139.6)	378.7 (154.5)	0.040

Radius of the circle single leg (mm):R_S_	22.4 (4.04)	18.2 (3.8)	0.028

Total excursion length single leg (mm):T_S_	1188.7 (435.2)	931.6 (159.1)	0.226

Anteroposterior excursion length single leg (mm):AP_S_	810.7 (410.9)	582.5 (134.2)	0.053

Mediolateral excursion length single leg (mm):ML_S_	668.1 (174.3)	586.0 (83.2)	0.083

Load distribution differences (%): LD	6.4 (6.7)	27.8 (15.9)	0.0005

Standing on two legs. The radius of the circle (R_D_) was 69.7% significantly greater in FFA compared with SPU(p = 0.0014). The length of total excursion (T_D_), the anteroposterior excursion (AP_D_) and the mediolateral length (ML_D_) during the two-leg task was, respectively, 41.5% (p = 0.071), 20.4% (p = 0.145) and 48.0% (p = (0.04) greater in FFA compared with SPU. The anteroposterior COP excursion was 29.4% greater than the mediolateral COP excursion in SPU (p ***), but almost identical in FFA (5.3% difference).

Standing on single (non-affected) leg. Radius of the circle (R_S_) was 18.7% less in FFA than in SPU (p = 0.028). FFA compared with SPU also showed 21.6% (p = 0.226), 28.1% (p = 0.053) and 12.3% (p = 0.083) less total COP excursion (T_S_), anteroposterior excursion (AP_S_) and mediolateral excursion (ML_S_) on single leg. The anteroposterior COP excursion was 21.3% greater than the mediolateral postural sway in SPU (p = 0.0124), but almost identical in FFA (0.6% difference).

### Comparison between double and single leg test variables

All postural stability variables were significantly greater standing on the non-affected leg compared with double leg stance in SPU (radius of the circle - 51.3%, p = 0.0018; total excursion - 60.6%, p = 0.0012; anteroposterior excursion - 59%, p = 0.0022; mediolateral excursion - 61.7%, p = 0.0037). In FFA the radius was almost identical when single and double leg stance was compared (difference: 1.6%). Total, anteroposterior and mediolateral excursion were significantly greater in single than double leg stance (p = 0.0025, p = 0.0015, p = 0.0041), but the differences in percentage (28.8%; 31.5%; 35,4%) were approximately half of those in SPU.

### Load distribution (LD) between the affected and non-affected leg

LD was significantly larger in FFA (27.8%) than in SPU (6.4%) (p = 0.0004). It should be noted, however, that the interindividual variability was high in both groups, especially in SPU.

Relationship between load distribution and postural stability variables.

LD asymmetry significantly correlated with the radius (r = 0.48, p <0.05), the total (r = 0.59, p <0.01), and ML sway (r = 0.60, p <0.01) only in FFA.

Slope of the regression line. The inclination angle of the regression line was 10.2(±13.3)º and 11.6(±6.4)º for SPU and FFA, respectively. For the left leg amputees, the regression line of the COP movements appeared mostly in the first and third quadrants while for the right leg amputees this line appeared in the second and fourth quadrants of the coordinate system (Figure 2).

The radius of the curve gave the best discrimination between the two groups as it was revealed by the results of discriminant function analysis below (Table 3). The number of variables in the model were 2, Wilks' Lambda was 0.39, F (2.16) = 12.49 at p < 0.0005.

**Table 3:** Discriminant function analysis for groups FFA and SPU

N = 28	Wilks'Lambda	Partial Lambda	F-remove (1.16)	Tolerance	1-Tolerance
R_D_	0.76	0.51	15.18	0.0012 *	0.14

R_S_	0.6	0.65	8.45	0.0102 *	0.14

* marked values indicate significant influence at p <0.05

To detect partial or spurious correlations between the predictor variables that could influence the interpretation of the discriminant function analysis, the relationship between R_D _and R_S _was but R_D _and R_S _were uncorrelated.

## Discussion

This study investigated the postural stability and balance strategy in unilateral lower-leg amputees due to vascular insufficiency. We hypothesized that there was an early adaptation in controlling balance because of the pain in the atherosclerotic leg that resulted in functional disturbances and alterations in balance control mechanism.

We hypothesized that there is an early adaptation in this patient population in contrast to trauma amputees because of the disease caused pain and functional disturbances [1]. Therefore we recruited so called fresh amputees being under prosthetic fitting process to test our hypothesis. Also we hypothesized that there has been a second adaptation process after familiarisation period and regular use of the prosthesis. It could be predicted that the two adaptation processes are bidirectional, namely in the early adaptation the body weight loads mostly the intact leg and on the contrary in the late adaptation the weight would be equally distributed on both non-affected and on the affected leg resulting in less postural sway.

We found that FFA distributed ~28% more of the total weight to the non-affected compared with the prosthetic leg. This result is similar to those published previously on FFA [14,15,26]. In contrast SPU, similar to healthy adults, distributed body weight to both affected and non-affected legs evenly [7,11,15]. In addition, as suggested by the large weight-bearing asymmetry in FFA and previous studies, postural sway was significantly greater in FFA than in SPU [13,9]. The significant correlations between load distribution and stability variables in FFA support this finding. However, the anteroposterior COP excursions were almost similar in the two groups, indicating a limited contribution from ankle muscles that control anteroposterior movement of the COP [27] and such movements are regulated mostly by proprioception which is absent in the amputated leg. The ankle in the amputated leg can be considered as a rigid, insensitive member of the kinematic chain. It was reasonable to assume that the normal balance strategy should have been changed to keep COP above the supporting area with the possible shortest trajectory over the prosthetic leg. The impairment in controlling anteroposterior excursion on the affected side which was the same in FFA and SPU resulted in increased mediolateral excursion COP trajectory and most probably the dominant COP motion direction did not coincide with the load/unload line. This finding agrees with the previous observations, i.e., the increased weight-bearing asymmetry causes reduced postural stability due to the large mediolateral sway.

We hypothesized that amputation patients with vascular insufficiency gradually shifted their body weight over the non-affected leg while standing or walking and learned a compensatory balance strategy which manifested in less postural sway while standing on the non-affected leg. We consider this shift in weight distribution as an early adaptation. Indeed, we found that the radius of the circle (R) containing 95% of COP trajectory was considerable reduced for FFA compared with SPU. The radius of the circle was the same in unilateral and bilateral standing for FFA which suggests of a modification in balance originated from the central and peripheral nervous system [13,28,29]. Therefore the circle of the curve discriminates the most accurately the two groups as revealed by the discriminant function analysis. However, we have to mention that the classification developed by the discriminant function was not tested with an independent sample of amputees.

However, there is a distinct difference in how we control balance while standing on one or two legs [27]: muscles that produce ankle eversion and inversion control mediolateral COP movements while standing on one leg but hip abductors and adductors control mediolateral sway while standing on two legs. Generally speaking balance control is task- and condition-dependent, resulting in compensatory strategies [30]. It is likely that organization of the different balance control strategies emerge independently. Consequently, when the task and conditions (internal and external) change, body sway adapts accordingly The duration of the adaptation to the altered circumstances in amputee patients is not known so far and therefore further studies are needed to be carried out.

Although our result may indicate a primary, compensatory adaptation we have to be to draw such a conclusion because of the limitation of our study. Namely, we did not study either patient with unilateral vascular sufficiency prior to amputation or trauma amputees who have no such adaptation possibilities due to the prompt loss of one of the limbs. However, previous studies may support our hypothesis. Mak and Ng [23] studied the effect of Tai Chi exercise on postural stability and found that regular training resulted in significant decrease in postural sway standing on single leg, but no significant change was found standing on double leg. Genthon et al. [19] concluded from their results that hemiparetic patients should learn a new balance strategy to improve postural stability accepting the remaining weight-bearing asymmetry. Also, the change in postural stability after finishing the familiarisation period and with regular use of prosthesis may indirectly support our assumption. Comparing the two groups we can recognize inverse relationship between the single leg and double leg postural stability variables. In FFA small postural sway standing on one leg was associated with increased postural sway standing on two legs. The SPU group demonstrated 2.2 times greater radius and 2.5-2.6 times larger COP excursion standing on one leg than on double leg.

We may speculate that new adaptive control mechanism might have been altered when they tried to load the prosthetic leg during double-leg stance, as indicated by the enlarged sway amplitude. In SPU the relationship is just the opposite. The data suggest that FFA patients feel more comfortable and safe to stand on one instead of two legs. We may conclude from this result that in the case of vascular first fitted amputees balancing on one leg is deemed to feel more safe than standing on two legs which most probably needs an adaptive alteration in balance strategy. This alteration may help the patients in the familiarisation process.

Our results suggest that during and after the familiarisation process patients gradually return to the normal balance strategy, namely the postural sway standing on both legs decreases because the patients are able to distribute weight almost symmetrically. Consequently postural sway increases while standing on one leg because the compensatory postural stability regulation learned before amputation loses its significance and patients feel safe to stand on two feet again. Our data do not fully agree with result by Vrieling et al [12] who concluded that rehabilitation should improve muscle strength and control in the non-affected leg to enable amputees to manage all postural stability disturbances rather than decreasing load-bearing asymmetry.

A limitation in the present study is that unlike Rougier and Genthon [31] we measured balance performance in amputees on one and not on two independent force platforms. We agree with Rougier and Genthon [31] that the traditional parameters do not provide sufficient information about ankle and hip strategy. Therefore we computed the dominant direction of the COP movements using a second order regression analysis on the stabilogram trajectory (equations 1 and 2) and calculated inclination angle (α) of the regression line (equation 3). The slope of the regression line shows the dominant direction of the COP movement, characterizing balance strategy (Figure 2). When α < 45º, the direction of the sway is predominantly lateral, and as a consequence, the subjects use mostly hip strategy. When α > 45º, the sway predominantly is in the AP direction, suggesting an ankle strategy. We observed relatively small α in both groups, suggesting predominantly mediolateral COP movements produced by hip strategy. Although the 31.6% difference between left and right leg amputees in α was not statistically significant, it seems that the regression line for right leg amputees pointed anteriorly toward the non-affected foot. Together, it seems that the second order regression analysis is sensitive tool to characterize balance strategies in amputees.

## Conclusion

In conclusion, large load distribution asymmetry in amputees produced small postural swaying while standing on one leg. This adaptation may occur before amputation. The role of this adaptation is to shorten the time needed to become familiar with the new prosthesis, hence it facilitates rehabilitation Finally, second order regression analysis is a sensitive method for characterizing balance strategies used by amputees.

## Competing interests

The authors declare that they have no competing interests.

## Authors' contributions

MA contributed to the design of the study, data collection, statistical analyses, and manuscript preparation. JT coordinated the study, contributed to the design of the study and to manuscript preparation. KB contributed to the design of the study, data collection, and manuscript preparation. ZC contributed to the design of the study and to the statistical analyses. EB contributed to the design of the study, data collection, and manuscript preparation. MH contributed to the design of the study and manuscript preparation. All authors read and approved the final manuscript.

## Pre-publication history

The pre-publication history for this paper can be accessed here:

http://www.biomedcentral.com/1471-2474/12/118/prepub
